# Ganetespib synergizes with cyclophosphamide to improve survival of mice with autochthonous tumors in a mutant p53-dependent manner

**DOI:** 10.1038/cddis.2017.108

**Published:** 2017-03-16

**Authors:** Evguenia M Alexandrova, Sulan Xu, Ute M Moll

**Affiliations:** 1 Department of Pathology, Stony Brook University, Stony Brook, NY 11794, USA

## Abstract

The DNA-alkylating cytotoxic agent cyclophosphamide (CTX) is commonly used in the clinic to treat hematological malignancies like lymphomas and leukemias as well as solid tumors, but shows dose-dependent potentially life-threatening toxicities and can induce secondary malignancies. Thus, the clinical utility of CTX would be improved if a companion drug could be identified that allows lowering the CTX dose, while maintaining or even increasing its antineoplastic therapeutic efficacy. In mouse models, high-dose CTX (300 mg/kg) is effective in treating T-lymphomas, while low dose (defined here as 100 mg/kg) is ineffective. We previously showed that the HSP90 inhibitor ganetespib potently suppresses T-lymphoma initiation and progression and extends overall survival (OS) in hotspot knockin mice expressing the p53 gain-of-function mutants R175H and R248Q (mutp53) by 30–59%. Here we asked whether ganetespib could potentiate the effect of low-dose CTX (100 mg/kg) in the autochthonous T-lymphoma-bearing mutp53 R248Q mouse model. Indeed, combinatorial CTX/ganetespib synergistically suppresses growth of autochthonous T-lymphomas in R248Q (p53Q/−) but not p53−/− control mice by reducing mutp53 levels and triggering apoptosis. Combinatorial treatment extends progression-free (PFS) and OS in p53Q/− mice significantly longer than in p53−/− mice. Specifically, PFS of p53Q/− mice improves 8.9-fold over CTX alone *versus* 3.6-fold in p53−/− mice. Likewise, OS of R248Q/− mice improves 3.6-fold, but worsens in p53−/− mice (0.85-fold) over CTX alone. Moreover, half of the p53Q/− mice on combinatorial treatment lived over 60 days, and one animal reached 121 days. In contrast, p53Q/− mice on single-drug treatment and p53−/− mice on any treatment lived less than 24 days. In sum, ganetespib synergizes with a sub-effective dose of CTX in mutp53 T-lymphomas by suppressing tumor growth and extending survival. Our results provide a potential strategy to reduce the effective clinical dose of CTX in mutant p53-bearing malignancies and attenuate CTX toxicity.

Cyclophosphamide (CTX) is one of the most widely used chemotherapeutics, effective in human lymphomas (e.g., aggressive non-Hodgkin's lymphomas and follicular lymphoma) and leukemias (e.g., small lymphocytic leukemia SLL and chronic lymphocytic leukemia CLL) as well as solid tumors (e.g., breast and ovarian cancer, bone and soft tissue sarcomas).^1^ Moreover, due to its immunosuppressive actions, CTX is also used in bone marrow transplantation conditioning and mobilization regimens and in autoimmune diseases.^2, 3, 4^ However, as DNA-alkylating agent CTX shows dose-dependent potentially life-threatening toxicities. High doses of CTX induce bone marrow suppression, cardiotoxicity^5, 6^ (which is dose-limiting), gonadal failure in males and females^7, 8^ and bladder toxicity (hemorrhagic cystitis and fibrosis),^9^ and CTX is associated with induction of secondary malignancies (bladder cancer, acute leukemia and skin cancer).^1, 10^ Thus, the clinical utility of CTX would be improved if a companion drug could be identified that allows lowering its dose while maintaining or even increasing its antineoplastic efficacy.

In mouse cancer models that express missense mutant p53, we and others previously showed that highly stabilized mutp53 gain-of-function proteins confer oncogenic addiction for tumor maintenance and metastasis.^11, 12, 13, 14, 15^ Conversely, mutp53 elimination significantly suppresses tumor growth and metastasis and markedly extends animal survival.^16, 17, 18^ Thus, in advanced autochthonous T-lymphomas genetic ablation of the human hotspot mutp53 R248Q allele triggers acute cancer cell apoptosis and suppresses tumor growth.^15, 16^ Moreover, early allele ablation at 10 weeks of age (at the stage of organ-confined incipient disease) prevents clinical lymphoma development in most animals and extends survival by 37%.^16^

p53 is mutated in ~50% of all human malignancies and in ~10% of hematological malignancies.^19, 20^ The HSP90 chaperone machinery is a critical determinant for mutant p53 stability by protecting the conformationally aberrant mutp53 proteins from degradation through their E3 ubiquitin ligases Mdm2 and CHIP.^21, 22, 23, 24^ Using the potent second-generation Hsp90 inhibitor ganetespib (Ganet, which showed a favorable safety profile in clinical trials),^25^ we found that Ganet triggers degradation of stabilized hotspot gain-of-function mutp53 R172H^11^ and R248Q^15^ *in vitro* and *in vivo*, accompanied by T-lymphoma apoptosis in both the allograft and autochthonous tumor setting.^16^ In clinical mouse trials, Ganet specifically extended survival of mutp53, but not p53null control mice, by 48% in the R175H cohorts and 59% in the R248Q cohorts.^16^ CTX given at high dose (300 mg/kg) was previously shown to be effective in inhibiting tumor growth of T-lymphomas in p53null mice.^26, 27^ Here we used the humanized (exons 4–9) mutp53 R248Q mouse model, which also primarily develops T-lymphoma, and report that Ganet synergizes with a low sub-effective dose of CTX to effectively suppress tumor growth and extend animal survival *in vivo*. This data might support a general strategy to reduce the effective dose of CTX without compromising efficacy in anticancer therapy of mutp53-expressing tumors.

## Results

**Figure 1:**
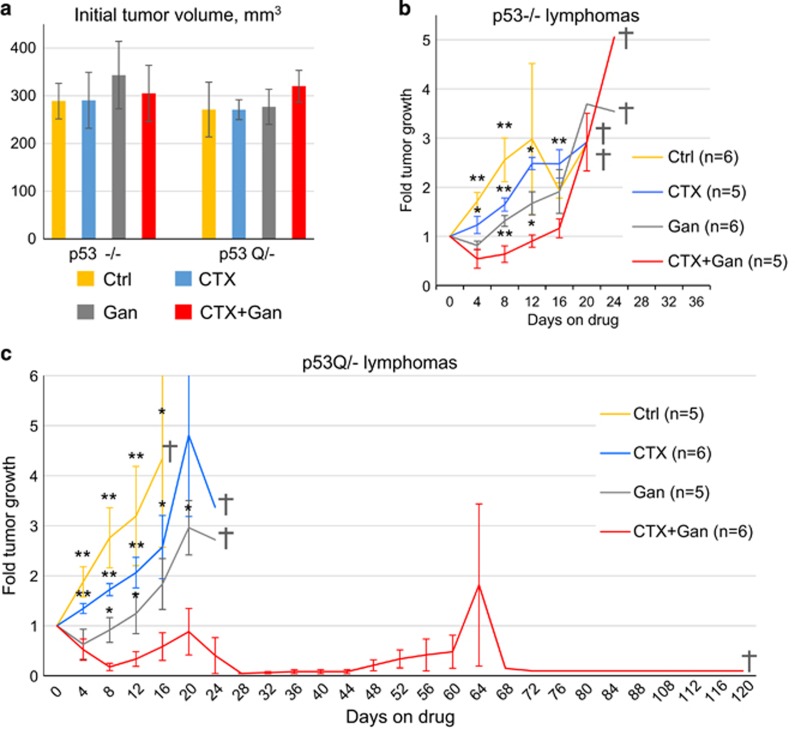
Treatment response of autochthonous T-lymphomas of p53−/− and p53Q/− mice, with or without single weekly doses of CTX alone (100 mg/kg), ganetespib alone (Gan, 50 mg/kg) or both drugs in combination (100 mg/kg CTX+50 mg/kg Gan). (**a**) Tumor volume at treatment start; mean±S.E.M. (**b** and **c**) Tumor growth over time normalized to initial tumor size in p53−/− (**b**) and p53Q/− (**c**) animals. Mean±S.E.M.; *n*, number of mice; gray crosses, day when last animal in the group was killed; *P<0.05, **P<0.01 relative to CTX+Gan

**Figure 2:**
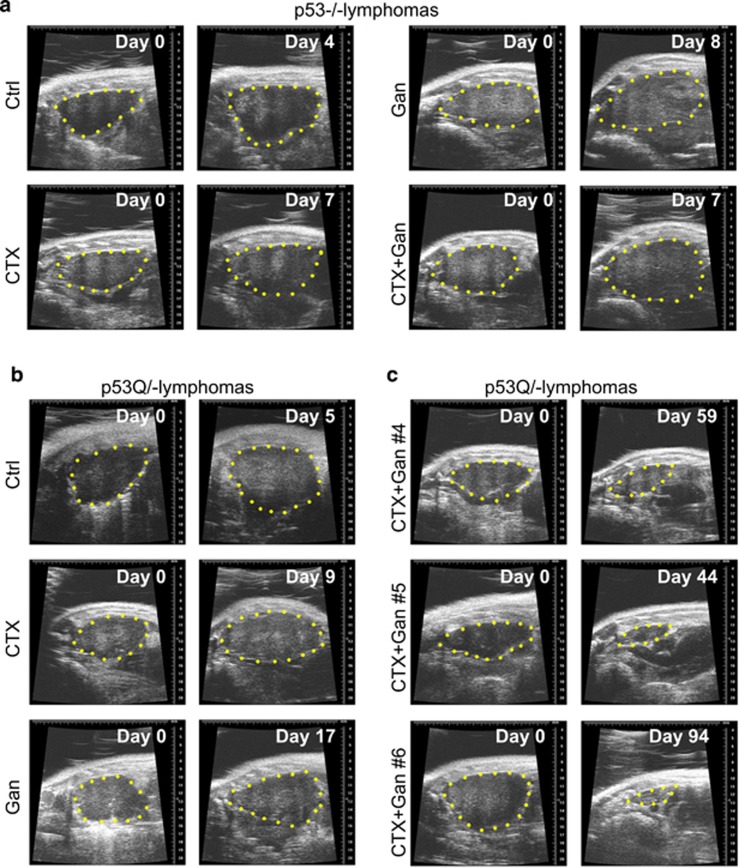
Representative high-resolution ultrasound images of autochthonous T-lymphomas of p53−/− and p53Q/− mice, with or without weekly treatment with CTX alone (100 mg/kg), ganetespib alone (Gan, 50 mg/kg) or both drugs in combination (100 mg/kg CTX+50 mg/kg Gan). (**a**) p53−/− lymphomas, (**b**) p53Q/− lymphomas, (**c**) p53Q/− lymphomas treated as indicated. Animal # refer to mice in Table 1

CTX given at high dose (300 mg/kg) was previously shown to be effective in inhibiting tumor growth of T-lymphomas in p53−/− mice.^26, 27^ To test the effect of combinatorial treatment of low-dose CTX plus Ganet on the growth of autochthonous T-lymphomas in p53Q/− *versus* control p53−/− mice and visualize tumor growth over time, we employed high-resolution ultrasound imaging.^16^ Thymus size and shape in p53Q/− and p53−/− control mice (about 40 per genotype) was examined starting at 8 weeks of age when the first T-lymphomas are known to arise.^15, 16^ When individual tumors had reached ~300 mm^3^ in volume (Figure 1a), each animal was randomly assigned to one of four treatment arms: (1) control (no treatment); (2) low-dose CTX alone (100 mg/kg i.p.); (3) low-dose Ganet alone (50 mg/kg i.v.) and (4) combined CTX (100 mg/kg i.p.) and Ganet (50 mg/kg i.v.). Mice were treated with the assigned drugs once a week, and lymphoma growth was monitored by sonography approximately every 4 days (Figures 1b and c and Figure 2). In agreement with our previous study,^16^ Ganet alone suppressed the growth of p53Q/− lymphomas to a greater extent than that of p53−/− lymphomas (Supplementary Figures 1a and b, gray *versus* yellow lines; Figures 1b and c), although this did not reach statistical significance (data not shown). Although with Ganet alone both p53−/− and p53Q/− lymphomas resumed their growth after a short regression/stagnation period (Figures 1b and c and Figures 2a and b), this time span was longer for p53Q/− *versus* p53−/− lymphomas (2 weeks *versus* 1 week, compare individual tumor growth in Supplementary Figures 1a and b), further confirming the previously observed mutp53-specific activity of Ganet.^16^ In contrast, due to the low-dose chosen, CTX alone suppressed tumor growth only mildly, and there was no difference in efficacy between p53−/− and p53Q/− lymphomas (Figure 1b, blue lines; Supplementary Figures 1c and d, blue lines; Figures 2a and b).

**Figure 3:**
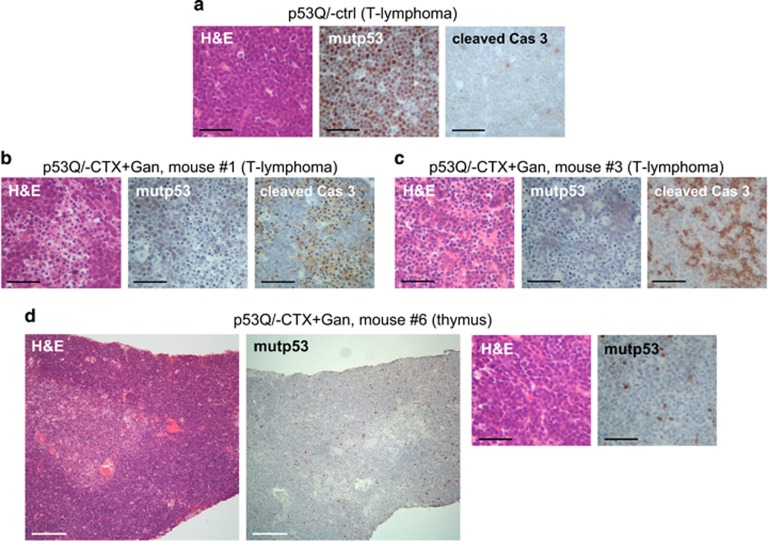
Representative T-lymphomas of p53Q/− mice treated with combinatorial CTX+Gan, taken at end point. mutp53 levels, apoptosis (cleaved caspase-3) and histopathology. (**a**) Untreated control tumor. Nearly 100% of tumor cells express highly stabilized mutp53. (**b**–**d**) Mice treated with combined CTX+Gan. (**b** and **c**) Combined treatment induced strong depletion of mutp53 protein in tumors accompanied by massive apoptosis. (**d**) With combined treatment, the initially large T-lymphoma (see Figure 2c) rapidly regressed within 7 days to a nearly undetectable thymic structure and remained like this until death. At autopsy, only normal thymus was detected, which contained rare isolated mutp53-positive cells. The animal had to be killed after 121 days due to a small mutp53-positive fibrosarcoma obstructing the anus (see Supplementary Figure 2a). Immunostaining with the indicated antibodies. H&E, hematoxylin and eosin. Black scale bar, 200 *μ*m; white scale bar, 800 *μ*m

Strikingly however, when CTX and Ganet were combined at their respective single doses, the growth of p53Q/− lymphomas was dramatically suppressed. Moreover, this combinatorial efficacy occurred preferentially in mutp53 tumors compared to p53−/− tumors (Figures 1b and c, red lines; Supplementary Figures 1c and d, red lines; Figure 2c). Growth suppression of p53Q/− tumors was accompanied by degradation of stabilized mutp53 and induction of apoptosis as indicated by hematoxylin and eosin and cleaved caspase-3 staining (Figure 3 and Supplementary Figure 2). Furthermore, while all untreated and single-drug-treated mice of both genotypes lived only up to a maximum of 24 days (Figures 1b and c; gray crosses indicate when the last mouse of the respective cohort died), half of the p53Q/− animals (three out of six) treated with CTX+Ganet – but none of the respective p53−/− animals – lived for more than 60 days after treatment start (Figure 1c, red lines; Supplementary Figure 1d, red lines; Table 1). One p53Q/− animal survived to 65 days with a small T-lymphoma but died of unknown cause (Table 1, mouse #5). Notably, the longest p53Q/− animal on CTX+Ganet (mouse # 6, Figures 2c and 3d) lived for 121 days with a near-complete cure of its T-lymphoma at autopsy. Pathology analysis of the mediastinum of this mouse by hematoxylin and eosin staining revealed only normal thymic tissue, with rare single mutp53-positive tumor cells sprinkled through the dense lymphoid tissue (Figure 3d). This animal had to be killed due to a small mutp53-positive fibrosarcoma that caused anal obstruction (Supplementary Figure 3a and Table 1). Taken together, with the notable exception of the p53Q/− cohort on combinatorial CTX+Ganet, pathological and immunohistochemical analyses showed that all other animals died from large recurrent T-lymphoma. These untreated control (Figure 3a) and single-drug-treated p53Q/− lymphomas (data not shown) exhibited highly stabilized mutp53. In contrast, only two out of six p53Q/− animals on combinatorial CTX+Ganet died from large T-lymphomas (mice #2 and #4, Table 1 and Supplementary Figure 3b). Two additional animals (mouse #1 and #3) died from interstitial pneumonia and lung atelectasis, respectively (Supplementary Figures 3c and d), and at autopsy had only small residual T-lymphomas with significantly reduced mutp53 levels and upregulated apoptosis (Figures 3b and c and Table 1). Taken together, these data indicate that the combinatorial CTX+Ganet treatment is much more effective in suppressing growth of mutp53 compared to p53null T-lymphomas by destabilizing mutp53 protein levels and triggering apoptosis. Moreover, in mutp53Q/− mice combinatorial treatment is also much more effective than CTX or Ganet alone.

**Figure 4:**
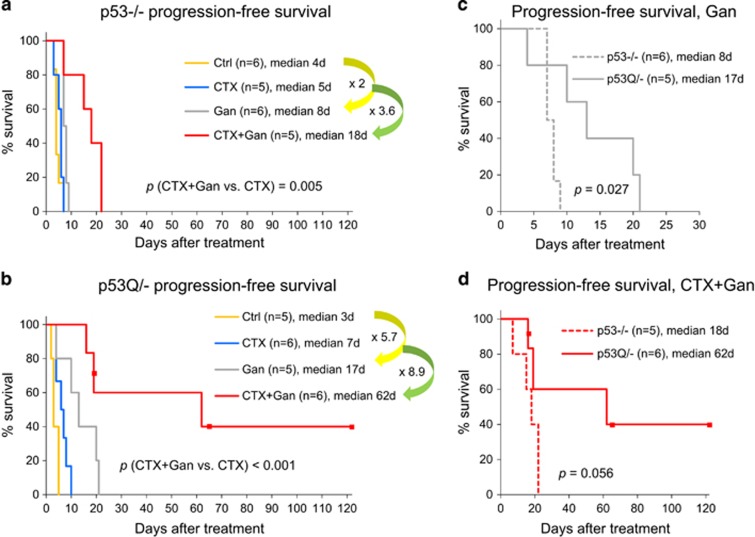
PFS of p53−/− and p53Q/− animals with T-lymphoma (initial tumor size about 300 mm^3^, see Figure 1), with or without weekly treatment with CTX alone (100 mg/kg), ganetespib alone (Gan, 50 mg/kg) or both drugs in combination (100 mg/kg CTX+50 mg/kg Gan). (**a**) p53−/− animals. (**b**) p53Q/− animals. (**c** and **d**) Paired p53Q/− *versus* p53−/− analysis of PFS of Gan-treated mice (**c**) and CTX+Gan-treated mice (**d**) of the indicated genotypes. Kaplan–Meier analysis; *n*, number of mice; *p*, log rank statistics. Dots represent censored animals because they died of reasons other than T-lymphoma

**Figure 5:**
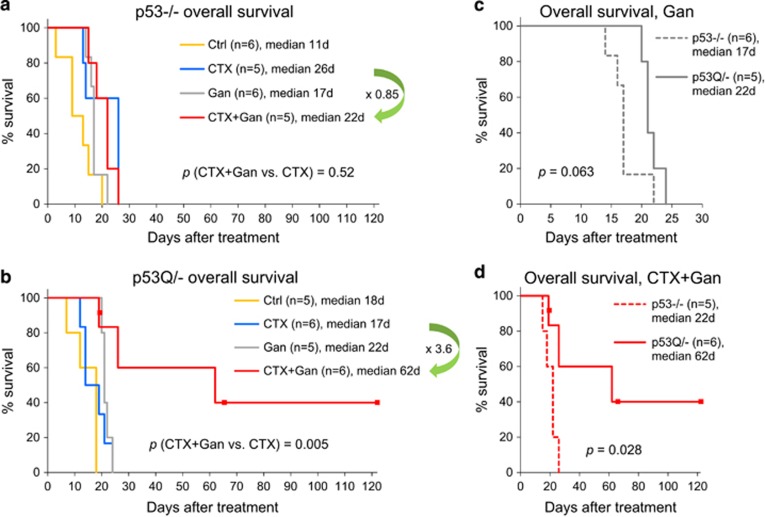
OS of the same p53−/− and p53Q/− animals with T-lymphoma as in Figure 4, with or without weekly treatment with CTX alone (100 mg/kg), ganetespib alone (Gan, 50 mg/kg) or both drugs in combination (100 mg/kg CTX+50 mg/kg Gan). (**a**) p53−/− animals. (**b**) p53Q/− animals. (**c** and **d**) Paired p53Q/− *versus* p53−/− analysis of OS of Gan-treated mice (**c**) and CTX+Gan-treated mice (**d**) of the indicated genotypes. Kaplan–Meier analysis; *n*, number of mice; *p*, log rank statistics. Dots represent censored animals

Analysis of progression-free (PFS) (Figure 4) and overall survival (OS) (Figure 5) demonstrated a superior potency of Ganet in p53Q/− *versus* p53−/− animals (Figures 4a–c, yellow arrows and Figure 5c), confirming our previous findings of a relative mutp53 specificity of Ganet treatment.^16^ In contrast, CTX had a similar – albeit none or minimal – efficacy in both genotypes (Supplementary Figure 4). Most importantly, however, combinatorial CTX+Ganet prolonged PFS and OS significantly longer in p53Q/− than in p53−/− animals. Compared to CTX alone, CTX+Ganet extended PFS and OS of p53Q/− animals 8.9-fold (from 7 to 62 days) and 3.6-fold (from 17 to 62 days), respectively (Figures 4b and 5b). In contrast, CTX+Ganet extended PFS of p53−/− animals only 3.6-fold (from 5 to 18 days) relative to CTX alone, and failed to extend their OS (in fact, OS decreased from 26 to 22 days, 0.85-fold) (Figures 4a and 5a). Thus, upon combined treatment median PFS was 3.4 times longer (62 *versus* 18 days, *P*=0.056, Figure 4d) and median OS was 2.8 times longer (62 *versus* 22 days, *P*=0.028, Figure 5d) in p53Q/− *versus* p53−/− animals. All together, these data demonstrate strong cooperation between low-dose CTX and Ganet in extending PFS and OS specifically in mutp53 tumors *in vivo*.

## Discussion

Here the gain-of-function hotspot mutant p53 R248Q mouse model, which primarily develops T-lymphomas, provided proof-of-principal that the Hsp90 inhibitor ganetespib potentiates the antineoplastic efficacy of CTX by suppressing autochthonous tumor growth and prolonging PFS and OS. These effects were significantly stronger in mutp53Q/− than in p53null mice, suggesting that the combinatorial treatment might specifically benefit patients with mutp53-bearing malignancies. One obvious explanation is that Ganet alone prolongs the survival of mutp53 – but not p53null – animals by destabilizing mutp53 and triggering its proteasomal degradation.^16^ Indeed, with Ganet-only treatment, we already saw stronger effects in mutp53 compared to p53null animals, indicated by a longer period of tumor stagnation from 1 week to 2 weeks (compare individual tumor growth in Supplementary Figures 1a and b, gray lines) and by significantly extended PFS from 2- to 5.7-fold (*P*=0.027, Figures 4a–c). There is also a trend for improved OS in mutp53 *versus* p53null mice (*P*=0.063, Figure 5c). A possible explanation why the differential effect of Ganet on mutp53 *versus* p53null animals was not quite as strong in this study as it was in our previous study^16^ is that previous treatment was started at 8 weeks of age at the stage of incipient disease with normal-sized thymus, while here treatment was started at the stage of advanced tumors (~300 mm^3^ in volume) at a median of 20 weeks (data not shown), shortening drug exposure. Moreover, in contrast to the previous study, the mutp53 *versus* p53null cohorts used here were not littermates.

Another possible explanation why combinatorial CTX+Ganet preferentially affects mutp53Q/− *versus* p53null tumors is that mutp53Q/− cancer cells proliferate faster than p53null cells, since growth advantage over simple p53 absence is one of the phenotypes of mutp53 gain-of-function.^15^ This could result in their higher sensitivity to the DNA-alkylating CTX. However, we did not see preferential sensitivity of mutp53Q/− mice *versus* p53null mice to CTX alone (Supplementary Figure 4). It seems that in combination, CTX and Ganet potentiate each other's efficacy and specificity toward mutp53-bearing tumors. It remains to be established whether CTX and Ganet will also synergize to suppress autochthonous tumors expressing different stabilized p53 missense mutants besides the hotspot R248Q described here. A potent and fast format for predicting efficacy against a whole spectrum of different p53 mutants is subcutaneous T-lymphoma allografts. Based on this, the likelihood for broader efficacy beyond R248Q is high, considering that CTX+Ganet synergism was also seen toward gain-of-function mutp53 R175H.^16^

Interestingly, treatment of either mutp53 or p53null animals with Ganet alone invariably results in tumor resistance and relapse (Figures 1a and b and Supplementary Figures 1a and b, gray lines). In human colorectal carcinoma cells, we previously described a Ganet resistance mechanism via elevated expression of UGT1A, a glucoronidating enzyme that causes excretion of Ganet into the extracellular space, eliminating its ability to inhibit intracellular Hsp90.^28^ However, this mechanism does not seem to be relevant in mutp53-driven T-lymphomas.^16^ Interestingly, however, in the combinatorial CTX+Ganet treatment arm, CTX completely prevented Ganet resistance in at least two out of six mutp53 animals (Table 1 and Supplementary Figure 1d, mice #5 and #6), further attesting to the mutp53-specific synthetic lethality of Ganet and CTX. While the mechanism of this synergistic effect and the generality of cytotoxic drugs preventing resistance to Ganet remain to be elucidated, our findings point toward the exciting possibility that the combination CTX+Ganet will facilitate both, to lower the effective CTX dose and side effects and to overcome resistance to Ganet.

## Materials and methods

### Mice and high-resolution ultrasound imaging

Hotspot humanized mutp53 R248Q mice and p53−/− control mice were previously described.^15^ Parental p53 R248Q/+ and p53−/+ strains, both on mixed 129SvImJ/C57Bl6J background, were intercrossed to obtain p53 R248Q/− and p53−/− mice. Animals were monitored weekly by ultrasound imaging, using the Visual Sonics Vevo 770 High-Resolution Imaging System. When tumors had reached 300 mm^3^ on average (ranging from 136 to 506 mm^3^), single weekly drug treatment was initiated as described below, followed by ultrasound imaging approximately every 4 days. Sagittal and transverse images were taken using the Vevo 770 V3.0.0 software. Tumor volume was calculated according to the formula for ellipsoid, *V*=*π*/6 × height × width × length. Mice were killed at end point, defined as appearing moribund. Note that the T-lymphoma size at end point was different for different animals, but on average was similar between treatment groups (Supplementary Figure 2a), except for p53Q/− mice treated with combined drugs, as explained in ‘Results' section. This variability is due to the fact that the clinical signs of the disease that require killing (labored breathing eventually leading to suffocation) correlate not only with tumor size, but also with tumor shape and compression effects on the cardio-vascular and pulmonary system depending on the specific location in the chest cavity. These parameters are variable and can cause the moribund state even when T-lymphoma size is not that enlarged. All animals were treated humanely and according to the guidelines issued by the Institutional Animal Care and Use Committee at Stony Brook University.

### CTX and ganetespib treatment

CTX (cyclophosphamide monohydrate, Sigma, C0768) was dissolved in phosphate-buffered saline and injected intraperitoneally at 100 mg/kg once a week. Ganetespib (STA-9090, Synta Pharmaceuticals, Lexington, MA, USA) was prepared as previously described^16, 29^ and injected into the tail vein at 50 mg/kg once a week. For combinatorial treatment, animals were injected with 100 mg/kg CTX plus 50 mg/kg ganetespib within 5 min of each other.

### Immunohistochemistry and histology

For immunohistochemical analysis, freshly dissected tissues were formalin fixed, paraffin embedded and sectioned (5 *μ*m). Slides were deparaffinized and boiled in citrate buffer (10 mM, pH 6.0, 35 min) for antigen retrieval, blocked in 10% goat serum and incubated with primary antibodies (mutp53, Santa Cruz FL393, Dallas, TX, USA, sc-6243, dilution 1 : 500; cleaved caspase-3, Cell Signaling (Danvers, MA, USA), 9661, dilution 1 : 500) for 2 h at room temperature. After PBS washing, slides were incubated with biotinylated secondary antibody and HRP-streptavidin using the Histostain SP Broad Spectrum kit (Invitrogen, Carlsbad, CA, USA, 959943B), stained with DAB substrate with hematoxylin counterstain and coverslipped. Other sections were stained by hematoxylin and eosin.

### Statistical analysis

Unpaired two-tailed Student's *t*-test was used to analyze tumor size, percent of apoptotic cells and percent of mutp53-positive cells. Kaplan–Meier analysis with log rank statistics was used to analyze PFS and OS. *P*<0.05 was considered statistically significant.

## Figures and Tables

**Table 1:** Response of p53 R248Q/− mice bearing autochthonous T-lymphomas to combinatorial treatment with CTX (100 mg/kg) and ganetespib (50 mg/kg)

**Mice treated with CTX+Ganet**	**OS after treatment start, in days**	**T-lymphoma growth at end point, relative to initial size**	**Cause of death**
Mouse #1^a^	19	1-fold	Interstitial pneumonia
Mouse #2	19	2.6-fold	T-lymphoma
Mouse #3	26	1.5-fold	Lung atelectasis, T-lymphoma
Mouse #4	62	3.4-fold	T-lymphoma
Mouse #5^a^	65	0.3-fold	Cause of death unknown. 11 days prior to death thymus still in regression (0.3-fold of original tumor size)
Mouse #6^a^	121	0.1-fold	Anal obstruction due to fibrosarcoma

a Censored in PFS and OS curves (Figures 4b and d and Figures 5b and d) since death was not due to T-lymphoma
