# Supplementary material for: Ganetespib synergizes with cyclophosphamide to improve survival of mice with autochthonous tumors in a mutant p53-dependent manner
Source: Cell Death Dis. 2017 Mar 16;8(3):e2683–. doi: 10.1038/cddis.2017.108 (PMC5386516; doi:10.1038/cddis.2017.108)
Supplement: Supplementary Information [file cddis2017108x1.ppt]

## Slide 1
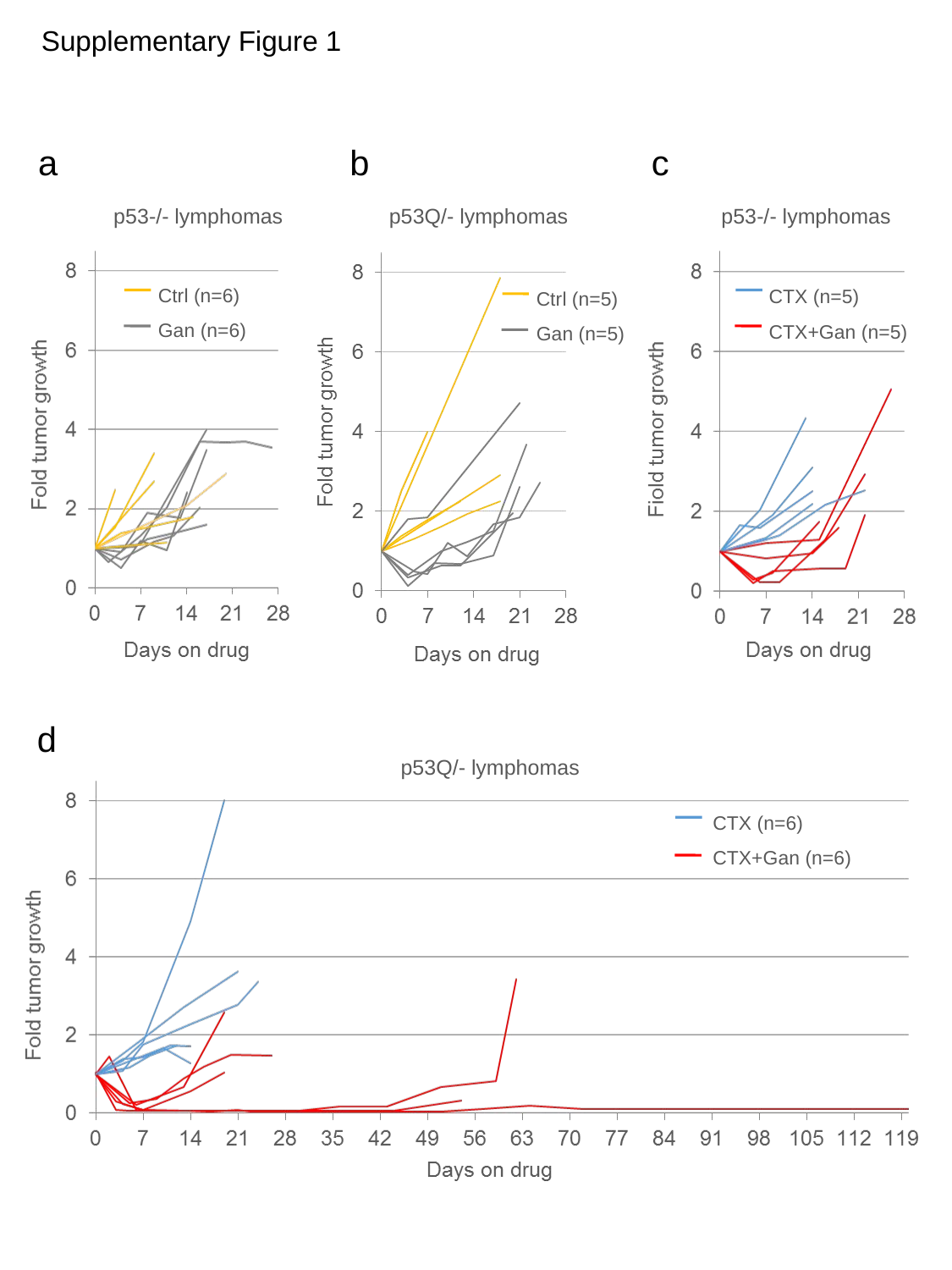

Supplementary Figure 1
a b c
p53-/- lymphomas p53Q/- lymphomas p53-/- lymphomas
Ctrl (n=6)
Gan (n=6)
CTX (n=5)
CTX+Gan (n=5)
Ctrl (n=5)
Gan (n=5)
d
p53Q/- lymphomas
CTX (n=6)
CTX+Gan (n=6)
