# Supplementary figures and images for: Ganetespib synergizes with cyclophosphamide to improve survival of mice with autochthonous tumors in a mutant p53-dependent manner
Source: Cell Death Dis. 2017 Mar 16;8(3):e2683–. doi: 10.1038/cddis.2017.108 (PMC5386516; doi:10.1038/cddis.2017.108)

## Slide 1
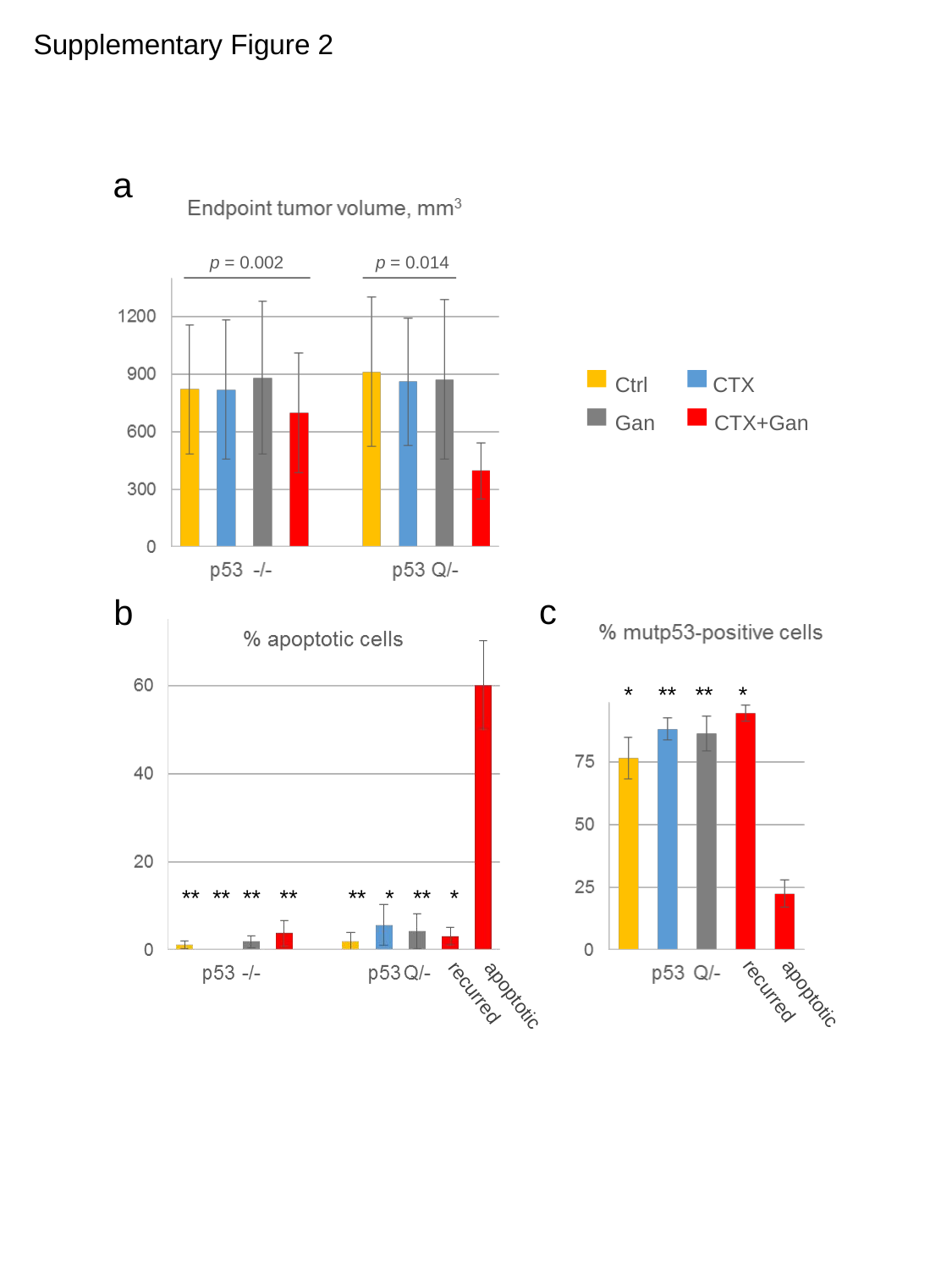

Supplementary Figure 2
a
p = 0.002 p = 0.014
Ctrl CTX
Gan CTX+Gan
** ** ** ** ** * ** *
* ** ** *
c
b
recurred
recurred
apoptotic
apoptotic

Supplement: Supplementary Information [file cddis2017108x2.ppt]

## Slide 1
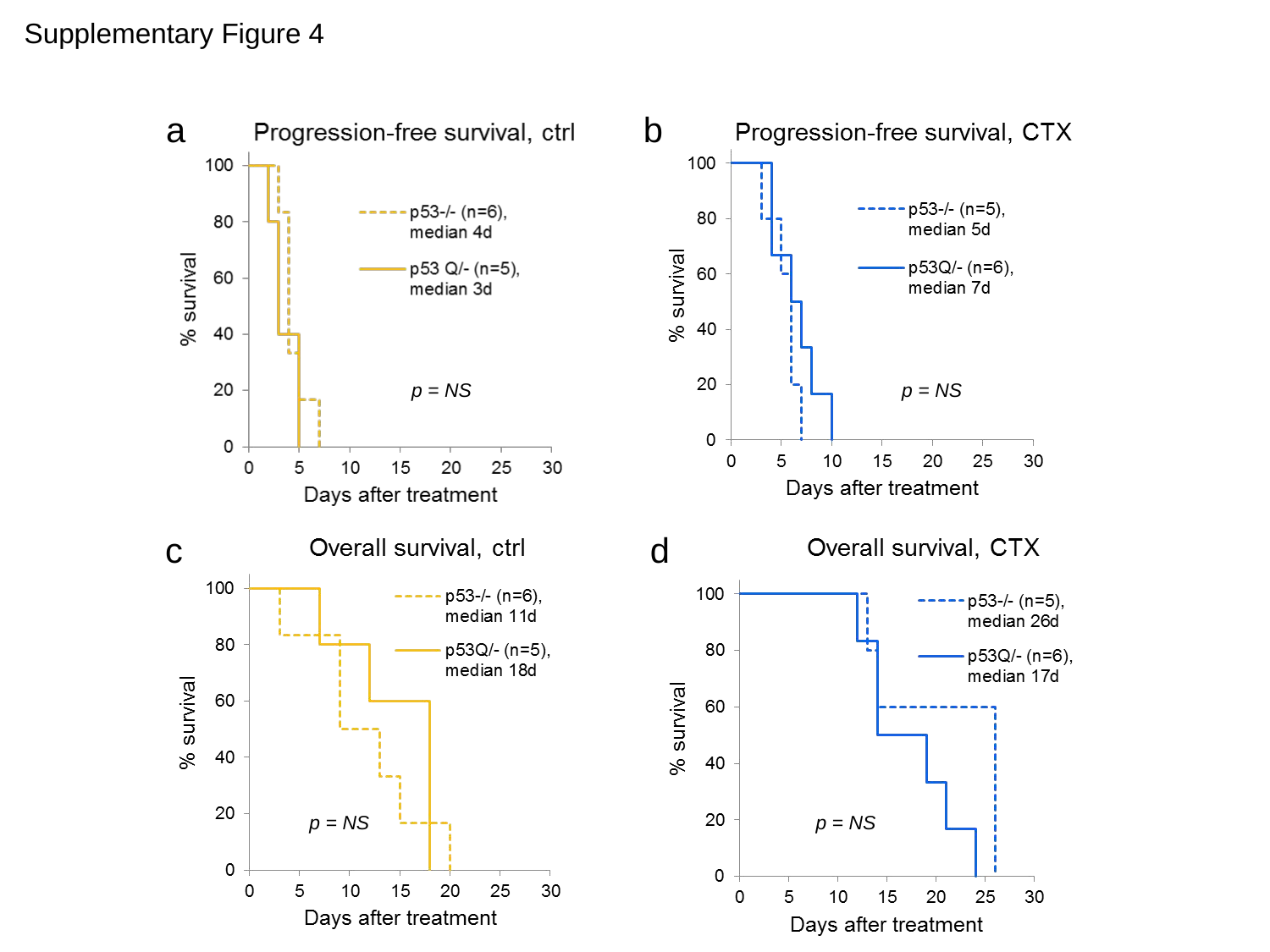

Supplementary Figure 4
a b
p = NS
p = NS
c d
p = NS
p = NS

Supplement: Supplementary Information [file cddis2017108x4.ppt]
