# Supplementary material for: Ganetespib synergizes with cyclophosphamide to improve survival of mice with autochthonous tumors in a mutant p53-dependent manner
Source: Cell Death Dis. 2017 Mar 16;8(3):e2683–. doi: 10.1038/cddis.2017.108 (PMC5386516; doi:10.1038/cddis.2017.108)
Supplement: Supplementary Information [file cddis2017108x3.ppt]

## Slide 1
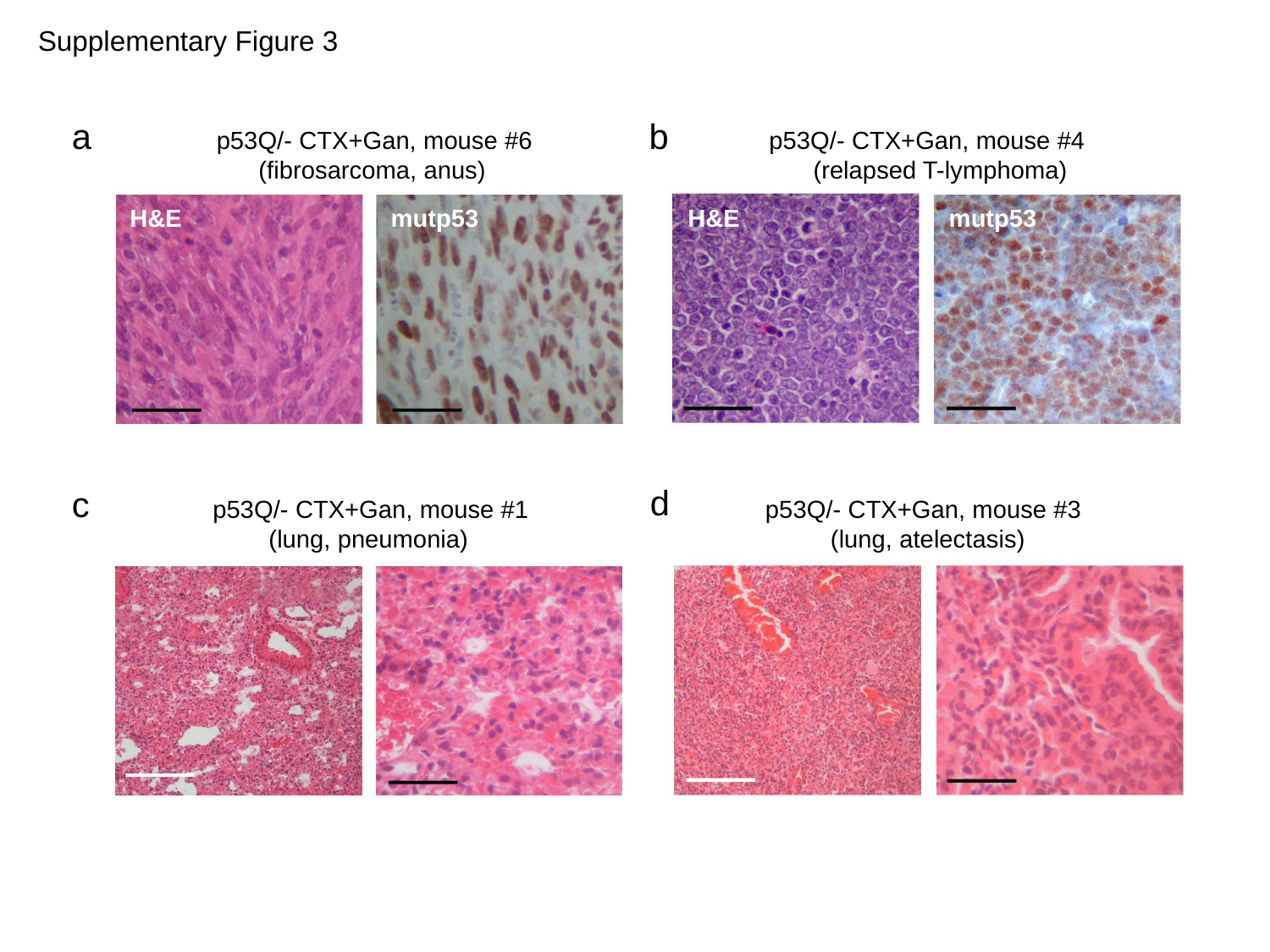

Supplementary Figure 3
a
b
p53Q/- CTX+Gan, mouse #6 p53Q/- CTX+Gan, mouse #4
 (fibrosarcoma, anus) (relapsed T-lymphoma)
H&E mutp53 H&E mutp53
H&E H&E H&E H&E
d
c
p53Q/- CTX+Gan, mouse #1 p53Q/- CTX+Gan, mouse #3
 (lung, pneumonia) (lung, atelectasis)
